# National and international collaborations to advance research into vascular contributions to cognitive decline

**DOI:** 10.1016/j.cccb.2023.100195

**Published:** 2023-12-14

**Authors:** Danit G Saks, Eric E Smith, Perminder S Sachdev

**Affiliations:** a Centre for Healthy Brain Ageing, Discipline of Psychiatry and Mental Health, School of Clinical Medicine, University of New South Wales, Sydney, New South Wales, Australia; b Department of Clinical Neurosciences and Hotchkiss Brain Institute, University of Calgary, Calgary, Alberta, Canada; c Neuropsychiatric Institute, Prince of Wales Hospital, Sydney, New South Wales, Australia

**Keywords:** Vascular cognitive impairment, Vascular dementia, Small vessel disease, Cerebrovascular disease, Collaboration, Consortia

## Abstract

Cerebrovascular disease is the second most common cause of cognitive disorders, usually referred to as vascular contributions to cognitive impairment and dementia (VCID) and makes some contribution to about 70 % of all dementias. Despite its importance, research into VCID has lagged as compared to cognitive impairment due to Alzheimer's disease. There is an increasing appreciation that closing this gap requires large national and international collaborations. This paper highlights 24 notable large-scale national and international efforts to advance research into VCID (MarkVCID, DiverseVCID, DISCOVERY, COMPASS-ND, HBC, RHU SHIVA, UK DRI Vascular Theme, STROKOG, Meta VCI Map, ISGC, ENIGMA-Stroke Recovery, CHARGE, SVDs@target, BRIDGET, CADASIL Consortium, CADREA, AusCADASIL, DPUK, DPAU, STRIVE, HARNESS, FINESSE, VICCCS, VCD-CRE Delphi). These collaborations aim to investigate the effects on cognition from cerebrovascular disease or impaired cerebral blood flow, the mechanisms of action, means of prevention and avenues for treatment. Consensus groups have been developed to harmonise global approaches to VCID, standardise terminology and inform management and treatment, and data sharing is becoming the norm. VCID research is increasingly a global collaborative enterprise which bodes well for rapid advances in this field.

## Highlights

• This paper summarises 24 large-scale collaborations into vascular contributions to cognitive impairment and dementia (VCID).
• Current research focuses on the mechanisms of action, means of prevention, and treatment of VCID.
• There have been previous and are ongoing consensus efforts focused on harmonising approaches for management of VCID and standardising terminology.
• Data sharing has become more common and accessible, using online data platforms such as Dementias Platform United Kingdom and Australia.
• The globalisation of VCID research is working towards increased awareness and understanding through large-scale multi-disciplinary collaborative efforts, which will inform future research and hopefully improve the management of VCID worldwide.

## 1 Background

It is estimated that cerebrovascular disease (CVD) is a contributing factor in about 70 % of dementia cases, while it is the major or only etiological factor in 15–25 % cases [1]. Vascular contributions to cognitive impairment and dementia (VCID), which covers the spectrum of cognitive impairment attributable to CVD, is therefore the second leading cause of dementia after Alzheimer's disease (AD) [2]. AD and CVD frequently co-occur in older people and have additive and possibly interactive effects [3,4]. Neuropathological features of CVD including white matter changes have also been shown to independently predict cognitive decline and dementia [5]. It is also recognised that VCID is amenable to prevention strategies since many of the vascular risk factors are eminently preventable [6]. Despite the promise of a preventable dementia that VCID offers, research into this disorder has lagged that into AD. There are several possible reasons for this discrepancy: the discourse around AD has captured the public's and funders’ imaginations such that AD has become synonymous with dementia; the focus in relation to CVD has been greatly on stroke and not the other vascular pathology contributing to cognitive impairment; VCID has a diverse set of underlying pathologies and mechanisms that lead to research fragmentation; and biomarkers for VCID have been slow to develop.

The time for a reset appears to have arrived. A major scientific statement on VCID was released in 2011 [7] and a significant translational ‘Think Tank’ on VCID met in 2015 [8], both examined the state of the science in this field. The National Institute of Health, USA, published a framework for advancing research in the cerebrovascular biology of cognitive decline in 2016 [9]. The World Health Organization recently published the Blueprint for Dementia Research [10] and identified VCID as an area requiring greater focus. A key recommendation from all these efforts was a greater need for large and collaborative effort. Researchers around the world appeared to have paid heed, and large number of national and international collaborations have emerged with a focus on VCID. In this paper, we survey some of the salient collaborations that are ongoing or have been recently completed, and examine their potential to contribute knowledge, develop interventions, provide data for sharing, and build capacity.

## 2 Method

**Fig. 1:**
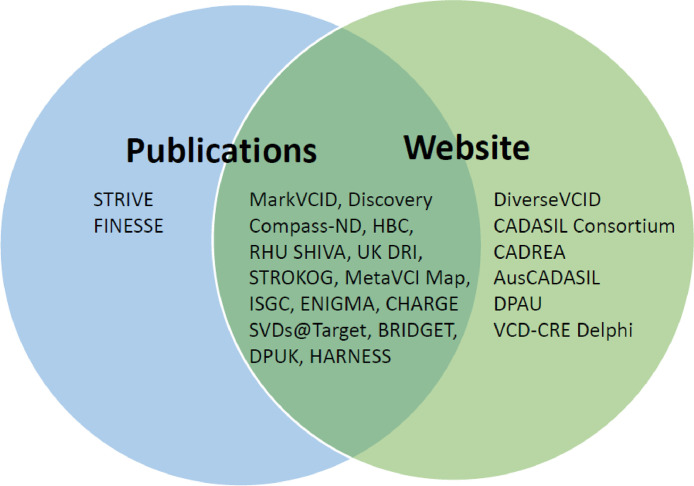
Venn diagram indicates that most collaborations have peer-reviewed publications (blue circle) and a website (green circle). Collaborations were identified by literature search (Pubmed, published 2015–2023) and by searching the web. Subsequent direct approach to principal investigators/key contacts in turn provided chain-referrals. Details of collaborations including location, year of establishment and sample size can be found in Supplementary Table 1.

Collaborations included were identified by literature search (Pubmed, published 2015–2023), direct approach to principal investigators/key contacts, and chain-referrals from these contributors. Initiatives were included if they met the criteria of 1) focused on VCID, 2) collaborative either national or international and 3) ongoing or recently completed. While this list cannot be considered to be exhaustive, the authors believe it represents a substantive overview of international collaborative research into VCID. The list will require periodic updating. Information included in this paper was obtained from direct communication with key contacts, publications and/or consortia websites. See Fig. 1 for avenues to obtain further information.

## 3 Collaborations to understand disease mechanisms and develop biomarkers for VCID

### 3.1 National collaborations

#### 3.1.1 Biomarkers for Vascular Contributions to Cognitive Impairment and Dementia (MarkVCID)

MarkVCID is a USA-based consortium that identifies and validates biomarkers involved in the pathophysiology underlying cerebral small vessel disease (cSVD)-related VCID. MarkVCID2 extends MarkVCID1 [11], [12], [13], to establish biomarkers for incorporation into cSVD-VCID clinical trials for subject selection and study outcomes. MarkVCID2 will enrol 1800 participants to complete clinical validation studies, prioritizing individuals with cognitive complaints and/or early impairment. Biomarker kits which will undergo clinical validation include MRI- and plasma-based measures [14], [15], [16], and combinations of MRI-and plasma-based measures. MarkVCID2 will categorise participants as progressed or non-progressed cSVD/VCID at the three-year follow-up visit and estimate sensitivity and specificity of baseline biomarker measures to identify future cSVD/VCID progression. The consortium will also analyse change in the candidate biomarkers for their validity as efficient outcome markers. Biomarker kits will be available for use in cSVD-VCID interventional trials to predict likelihood of worsening of the vascular component of VCID, and to streamline trials to test novel vasculoprotective treatments.

#### 3.1.2 Diverse Vascular Contributions to Cognitive Impairment and Dementia (DiverseVCID)

The impact of vascular disease on dementia risk may be exacerbated in African/Black and Hispanics/Latino Americans, who are at greater risk for vascular disease [17] and for whom vascular disease may have greater impact in dementia [18], [19], [20]. The USA-based DiverseVCID project aims to recruit 2250 diverse at-risk older Americans with subjective cognitive complaints, to participate in a 6-year study involving cognitive assessment, blood analysis (DNA and biomarkers) and neuroimaging. The goals of this study are to: (1) identify the extent and characteristics of white matter injury that influence cognitive and health outcomes; (2) evaluate mechanisms of progression of white matter injury on cognition and health outcomes; and (3) build and validate a predictive risk model for patients with white matter lesions to improve precision medical management and planning, for clinical care and inclusion criteria for future therapeutic studies.

#### 3.1.3 Determinants of Incident Stroke Cognitive Outcomes and Vascular Effects on RecoverY (DISCOVERY)

The USA DISCOVERY Network investigates mechanisms of susceptibility and resilience to post-stroke cognitive impairment (PSCI) and dementia to develop potential targets for personalized medicine and reduce post-stroke burden [21]. This prospective, multi-centre, observational collaboration is enrolling 8000 ischemic and haemorrhagic stroke patients without dementia during their acute hospital admission for two-year minimum follow-up. Participants will undergo serial cognitive evaluations and functional post-stroke assessments, while subsets of participants will additionally undergo research-based MRI, positron emission tomography scans, genetic/genomic and fluid biomarker testing.

#### 3.1.4 Comprehensive Assessment of Neurodegeneration and Dementia (COMPASS-ND)

The Canadian COMPASS-ND cohort study aims to discover and validate new risk factors and biomarkers of neurodegenerative disorder progression [22]. COMPASS-ND has enrolled 1772 individuals with memory concerns (153 VCID and 108 mixed dementia including VaD). Participants underwent comprehensive neurological and neuropsychological (follow-up at two years) assessment, and completed questionnaires on diet, lifestyle habits, social networks and caregiving, as well as objective vision and hearing assessment, research brain-MRI, and biospecimen collection (blood, urine, saliva, and stool). Plasma amyloid beta and phosphorylated tau testing is underway. A comprehensive online database of risk factors, clinical measures, blood analyte measures and MRI outcomes has been created for use by external researchers. An initial evaluation (200 subjects) found that covert cerebrovascular disease on neuroimaging was common in many of the cognitive disorders [23].

#### 3.1.5 Heart Brain Connection (HBC)

The Dutch HBC consortium was developed to explore the role of haemodynamic abnormalities along the heart-brain axis in VCID including the aetiology, assessment, and management of VCID with roots in clinical care [24]. HBC1 has been extended into HBC crossroads (HBCx) which is addressing additional haemodynamic factors, including blood pressure, cerebrovascular reactivity, valvular, rhythm, and endothelial abnormalities. Epidemiological, clinical, and autopsy studies have shown that haemodynamics and cardiovascular disease in VCID [25], [26], [27] need to be considered in the context of common comorbidities, in particular AD. HBCx have explored cerebral amyloid in patients with cardiovascular disease [28], to inform the AMYCODE study. HBC is addressing cerebral haemodynamics and cognition in patients undergoing transcatheter aortic valve implantation [29] in the CAPITA study. In cSVD, HBC have supported evidence of blood pressure pattern variability; cardiac and aortic measures of hypertensive exposure on cardiovascular MRI also related to cognitive impairment [30]; small vessel visualisation with 7T MRI; and coagulation blood-based biomarkers. In November 2022, the first Heart Brain Clinic formally opened at the Amsterdam University Medical Centre.

#### 3.1.6 RHU SHIVA

The French RHU SHIVA consortium combines academic and clinical experts with industry partners to focus on VCID resulting from cSVD under three main themes: (1) diagnosis stratification; (2) molecular mechanisms; and (3) therapeutic implications. The consortium is organised into six non-discrete cSVD work packages including imaging biomarkers for diagnosis and characterization; multiomics biomarker signature; biological mechanisms and putative biotargets; personalised early risk prediction of cSVD complications; preventative management and ethical implications; and project management and dissemination. SHIVA, in conjunction with the CHARGE consortium, recently published the first genomic study on perivascular space burden, as a marker of cSVD, providing novel insight into the significance of perivascular space and potential for therapeutic avenues [31]. SHIVA also prioritises education, having hosted seven scientific mini symposia to-date.

#### 3.1.7 UK Dementia Research Institute (UK DRI) Vascular Theme

The UK DRI involves 750 researchers and over 50 support staff to investigate dementia-related neuropathologies. The UK DRI Vascular Theme focuses on vascular contributions to dementia, e.g., mechanisms behind vascular and blood-brain barrier (BBB) dysfunction and the role of glial cells in vascular dysfunction. The Vascular Theme also prioritises education on vascular causes of neurodegeneration through meetings and workshops on research strategy, priorities, and approaches, and through the national early career researcher network, managed by UK DRI. The Vascular Theme searchable database includes 14 potential vascular models for dementia research, with input from DPUK and others, expected to be accessible in early 2024. Methods for monitoring early detection were piloted in the *Rates, Risks and Routes to Reduce Vascular Dementia* (R4VaD) study [32]. R4VaD also generated the ordinal cognitive assessment used in the LACunar Intervention Trial 2 (LACI-2 [33]) to demonstrate benefits of repurposed vascular drugs in reducing VCI. Examples of Vascular Theme member publications, in collaboration with others include studies of cSVD [31,34] and dementia [35], [36], [37].

Table 1 indicates for each collaboration, the year of establishment, whether it is national (multi-site) or international, and website address, if applicable.

**Table 1:** National and international collaborations.

**National Initiatives**	**Year**	**Country**	**Website**

MarkVCID	2017	USA	https://markvcid.partners.org/
DiverseVCID	2020	USA	https://diversevcid.sf.ucdavis.edu/
Discovery	2019	USA	https://discoverystudy.org/
COMPASS-ND	2018	Canada	https://ccna-ccnv.ca/compass-nd-study/
HBC	2013	The Netherlands	http://www.hart-brein.nl/
UK DRI Vascular Theme	2019	UK	www.ukdri.ac.uk
CADASIL Consortium	2022	USA	https://cadasil-consortium.org/
AusCADASIL	2023	Australia	https://cheba.unsw.edu.au/research-projects/vascular-contributions-dementia-centre-research-excellence/auscadasil
VCD-CRE Delphi	2022	Australia	https://cheba.unsw.edu.au/research-projects/vascular-contributions-dementia-centre-research-excellence/delphi
RHU-SHIVA	2020	France	https://rhu-shiva.com/en/

**International Initiatives**	**Year**	**Regions**	**Website**

Meta VCI Map	2017	Europe, Australasia, Americas	https://metavcimap.org/
STROKOG	2016	Europe, Australasia, Americas, Africa	https://cheba.unsw.edu.au/consortia/strokog
DPUK	2014	Europe, Australasia, Americas, Africa	https://www.dementiasplatform.uk/
DPAU	2021	Europe, Australasia, Americas, Africa	https://www.dementiasplatform.com.au/
CADREA	2023	East Asia	-
STRIVE	2012	Europe, Australasia, Americas, Africa	-
FINESSE	2020	Europe, Australasia, Americas	-
HARNESS	2018	Europe, Australasia, Americas	www.harness-neuroimaging.org
ISGC	2007	Europe, Australasia, Americas, Africa	www.strokegenetics.org
ENIGMA Stroke Recovery	2009	Europe, Australasia, Americas	https://enigma.ini.usc.edu/ongoing/enigma-stroke-recovery/
VICCCS	2013	Europe, Australasia, Americas, Africa	-
SVDs@Target	2016	Europe, Americas	https://www.svds-at-target.eu/index.html
BRIDGET	2016	Europe, Australasia, Americas	https://bridget.u-bordeaux.fr/
CHARGE	2009	Europe, Americas	https://web.chargeconsortium.com/

### 3.2 International collaborations

**Fig. 2:**
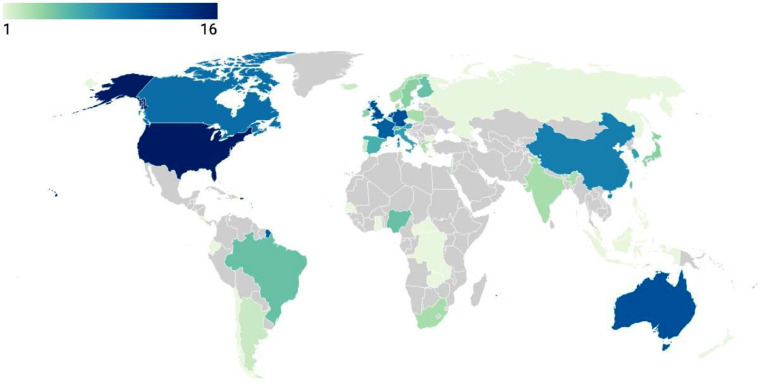
Countries involved in the collaborations listed in this paper. The figure indicates the underrepresentation of low- and middle-income countries in global collaborative efforts. Figure created with DataWrapper.

A significant challenge in research is obtaining large-enough cohort sizes to address the gaps in scientific knowledge. International consortia, supported by data-sharing platforms act as a means of amalgamating similar studies to pool datasets to enable powerful statistical analyses and meaningful output. These consortia often allow access for relevant researchers to obtain subsets of data upon justifiable request. They also promote a collaborative research community and facilitate capacity building in less resourced environments. The distribution of collaborations is visually represented in Fig. 2.

#### 3.2.1 Stroke and Cognition Consortium (STROKOG)

STROKOG is an international collaboration of post-stroke/ transient ischemic attack or high vascular risk studies with cognitive decline or impairment as an outcome. STROKOG was established to harmonise data [38] and to conduct joint analyses on VCID. STROKOG aims to identify risk and protective factors for VCID across geographical regions and ethnic groups, with the intent that STROKOG findings help guide and optimize preventative strategies and health policy internationally. STROKOG currently includes 38 study cohorts from 18 countries and 5 continents. STROKOG data have shown that 44 % of stroke survivors in hospital-based stroke cohorts had impairment in global cognition [39]; stroke survivors experience faster cognitive decline than stroke-free controls from 1 to 3 years after onset [40]; and diabetes but not prediabetes is associated with poorer cognitive performance in patients 3 to 6 months post-stroke [41]. From 2023, STROKOG will also include intervention studies that aim to reduce the impact of cerebrovascular disease.

#### 3.2.2 Meta VCI map

The Meta VCI Map international consortium is a collaborative platform developed for meta-analyses on strategic lesion locations for VCID. The platform integrates neuroimaging data on vascular brain injury, in particular infarcts and white matter hyperintensities (WMH), and cognitive data from large multicentre population-based and cohort studies [42]. Meta VCI Map projects include infarct lesion symptom mapping, involving 13 cohorts, including over 3000 patients with ischemic stroke and cognitive testing within 15 months post-stroke. This led to the first comprehensive map of strategic infarct locations associated with risk of PSCI [43]; sex differences in PSCI [44]; WMH burden impact; and other multimodal prediction studies, also considering the role of disconnection [45]. Memory clinic studies of strategic WMH locations involving 11 cohorts (over 3500 patients) with MRI and cognitive testing have led to findings suggesting the WMH impact on cognition is location-dependent, primarily involving four strategic white matter tracts [46], as well as projects considering WMH distributions and aetiology. Finally, population studies of strategic WMH locations involving 15 cohorts (14,876 individuals) with MRI and cognitive data are currently being analysed.

#### 3.2.3 International Stroke Genetics Consortium (ISGC)

The ISGC was created by 16 investigators, with the aim of progressing stroke genetics research, through working groups, educational proceedings and supporting research. Currently, research supported by the ISGC focuses on the genetics, physiology, and outcomes of stroke including cognition and functioning. The ISGC hosts 6-monthly international workshops and has expanded to include over 200 stroke genetic researchers from approximately 40 countries (6 continents), with investigators and participants from diverse backgrounds. Projects developed and supported by the ISGC include MEGASTROKE, GIGASTROKE, GISCOME, GENISIS, and MRI-GENIE. The ISGC is structured into working groups such as the Cognitive Working group, established in 2019 with the aim of advancing understanding of the genetic underpinnings of PSCI and VCID. Key projects include Ibiostroke and CANVAS and will include data from DISCOVERY. Notable publications include the identification of shared genetic risk for ischaemic stroke and AD [47], genome-wide association studies [48,49] and meta-analyses identifying genetic determinants of stroke risk to inform drug targets [50].

#### 3.2.4 Enhancing Neuro Imaging Genetics through Meta Analysis (ENIGMA)- Stroke Recovery

The ENIGMA consortium brings together neuro-genetic researchers within 50 working groups including Stroke Recovery. ENIGMA-Stroke aims to improve understanding of post-stroke brain changes relative to functional outcomes and recovery [51] by: (1) creating a worldwide network of stroke neuroimaging centres focused on understanding neural mechanisms of stroke recovery; (2) computing and analysing metrics of brain shape, volume, wiring and function post-stroke; (3) identifying structural and functional differences in post-stroke brain outcomes and exploring the relation between these measures and functional outcomes and/or recovery and rehabilitation; and (4) developing collaborations and infrastructure for novel stroke brain-behaviour analyses. ENIGMA-Stroke have released open-source datasets where permissible by local ethics boards, such as a recently shared dataset of 1279 stroke MRIs with manually segmentation lesion masks [52]. Although initial work focused on post-stroke sensorimotor outcomes [53], [54], [55], current research explores neural associations with post-stroke on cognition, mood, and language as well as the effects of therapeutic approaches, exercise and sleep on stroke outcomes.

#### 3.2.5 Cohorts for Heart and Aging Research in Genomic Epidemiology (CHARGE) consortium

The international CHARGE consortium was formed to facilitate genome-wide association study meta-analyses and replication opportunities among multiple large and well-phenotyped longitudinal cohort studies [56]. CHARGE is responsible for 262 publications since its inception. CHARGE is structured around approximately 40 working groups. The neurology group (NeuroCHARGE) coordinates large consortia to investigate stroke, dementia including biomarkers, cognition function, and neuroimaging outcomes.

#### 3.2.6 SVDs@target

The SVDs@target international collaboration, coordinated by Ludwig-Maximilians-University Munich, Germany, was established to identify mechanisms of SVD and validate these mechanisms through intervention, ultimately with the goal of reducing cSVD burden and preventing stroke and dementia. SVDs@target used animal models and human subjects to assess blood pressure variability and microvessels; BBB integrity and perivascular flow; microvascular matrisome and vascular integrity; inflammatory mechanisms and validated these findings through interventions in animal models and patient cohorts. Some key findings have included novel neuroimaging markers of cSVD using 7 Tesla MRI [57], collocation of SVD brain lesions with high BBB leakage [58], and implications of focal vessel-clusters in white matter identified using susceptibility-weighted imaging in cSVD [59].

#### 3.2.7 BRain Imaging, cognition, Dementia and next generation GEnomics: a Transdisciplinary approach to search for risk and protective factors of neurodegenerative disease (BRIDGET)

BRIDGET is an international collaborative effort, led by the University of Bordeaux, of research into neuroimaging, cognition and genomics of brain ageing. This was separated into two task forces in key domains: genomic and epigenomic analysis, and neuroimaging. These task forces operated three work packages: (1) identify genetic variants associated with structural makers of brain ageing; (2) explore lifetime determinants of brain ageing via longitudinal profiling of genomic, epigenomic and environmental markers; and (3) clinical and functional significance of genetic determinants for structural brain ageing, with a focus on cSVD. BRIDGET has now been completed; however, the data collected are still being analysed in conjunction with ongoing initiatives. Some key BRIDGET publications have identified loci for WMH volume in older adults [60], suggested distinct causes for periventricular and deep WMH [61], and indicated novel genetic variants in Alzheimer's disease which are involved in the immune response and transcriptional regulation [62].

### 3.3 Disease-specific collaborations

There is growing interest in the most common monogenic form of cSVD, Cerebral Autosomal Dominant Arteriopathy with Subcortical Infarcts and Leukoencephalopathy (CADASIL) [63]. The following emerging initiatives focus on CADASIL as a model for investigating VCID.

#### 3.3.1 CADASIL Consortium

This North America-based CADASIL Consortium is recruiting a longitudinal cohort of 400 adult participants with CADASIL *NOTCH3* mutations (family history and/or genetic testing) and 100 non-carrier controls. Clinical, neuroimaging, and molecular phenotyping, including AD biomarkers, will be acquired across twelve sites with established CADASIL clinics to characterize the biological and clinical course from the pre-symptomatic stage through dementia. The CADASIL consortium aims to replicate previous findings of specific *NOTCH3* mutations which manifest more severe CADASIL [64], and other findings, to help provide families with accurate prognostics. Additionally, the Consortium aims to provide standardization of CADASIL methods and measures for worldwide collaboration. Computerized assessments will be implemented to facilitate cross-site reliability for future large collaborative rare disease studies. All biofluids will be stored for current and future biomarker discovery and validation studies. Next-generation genomic analyses will be shared for ongoing advancement of VCID to better understand lifestyle and environmental contributions to outcomes in cerebrovascular diseases.

#### 3.3.2 CADASIL Registry in East Asia (CADREA)

CADASIL is known to vary in its symptom profile and severity in relation to the specific NOTCH3 mutation present. Notably, some mutations, such as *NOTCH3* p.R544C and p.R75P are seen only in East Asia. CADASIL with the p.R544C mutation (0.9 % in general population) usually does not cause migraine [65] and the p.R75P mutation (20 % of Japanese CADASIL) have been reported to not show white matter lesions in the temporal pole, considered a specific imaging finding in Western patients [66]. In Japan, a phase II study, the AMCAD (Adrenomedullin for CADASIL), has been conducted with 60 CADASIL patients and the results are currently being analysed, but moving to large-scale phase III poses challenges. The East Asian CADASIL cohort (CADREA) formed by researchers in Japan, Korea and Taiwan [67] will aid in appropriate diagnosis and prognosis of CADASIL and the development of future treatment options. This consortium aims to recruit 1000 individuals to accumulate longitudinal data on the genotype and phenotype (cognitive function, imaging findings) of CADASIL patients, which will form the basis for future pivotal studies.

#### 3.3.3 AusCADASIL

To date there have been no large-scale Australian studies of CADASIL. The AusCADASIL collaboration was recently established to examine the clinical features and longitudinal course of CADASIL. This cohort will acquire clinical, neuroimaging, blood, and retinal phenotyping and extensive neuropsychological profiling to determine early markers and progression of CADASIL. This study also aims to determine the pathogenic variants in the *NOTCH3* gene in Australian patients, and the influence of different spectra of *NOTCH3* variants on the clinical phenotype of CADASIL. AusCADASIL utilises a multidisciplinary team with varied expertise to contextualise the findings within the Australian health system. The study will be completed across five centres in three states in Australia with an anticipated 150 *NOTCH3* positive individuals (confirmed CADASIL, suspected CADASIL-either *NOTCH3* positive or symptomatic) and equivalent *NOTCH3* negative healthy controls without cognitive decline. This study also aims to serve as a resource for CADASIL research in Australia by providing educational materials for participants, carers, and family members. AusCADASIL will store fluid samples in the Centre for Healthy Brain Ageing Research BioBank for further future analysis.

### 3.4 Data-sharing platforms

To extend the benefit of research beyond the investigators involved in a study or a collaboration, sharing of data with external researchers is increasingly supported by funding bodies and the investigators themselves. Several platforms have been developed to facilitate this process and promote easy and equitable access, while protecting privacy. While none of these platforms is exclusively for VCID, two platforms that support VCID studies are listed. Data-driven collaborative efforts are listed in Table 2.

#### 3.4.1 Dementias Platform United Kingdom (DPUK)

DPUK is a data-driven platform [68] that convenes experts in academia, pharmaceutical industry, and charities to improve dementia detection, treatment and prevention by providing access to findings, technology, and volunteers [69,70]. DPUK prioritises collaboration, with strong links to its Australian counterpart- DPAU -, as well as the Korean Dementia Research Centre and the Alzheimer's Disease Data Initiative (ADDI). To date there have been 1500 outputs from DPUK activities including: 26 academic, industry and third sector partners with over 1000 cohort access requests resulting in 250 research publications including over 50 cohort studies and research data for 3.5 m people. DPUK continues to accelerate progress in research on all types of dementia, including VCID, and support the translation of basic science into practice through three main pathways: repository of dementia-optimised cohort data (DPUK Data Portal), engine for matching public volunteers to the most appropriate new research studies (the Trials Delivery Framework), and programme of cutting-edge experimental medicine (the Experimental Medicine Incubator). Recently, DPUK in conjunction with the UK DRI and the British Heart Foundation, addressed the shortfalls of understanding of VCID being largely driven by limited VCID models and studies, and outlined recommendations for improving future research [71].

#### 3.4.2 Dementias Platform Australia (DPAU)

DPAU is a data sharing platform led by the University of New South Wales Centre for Healthy Brain Ageing (CHeBA), established with Monash Secure eResearch Platform and DPUK. DPAU hosts data from international longitudinal and cross-sectional studies of brain ageing and enables researchers to explore and identify relevant studies, apply for data access, and analyse data in a secure, remote environment. DPAU enhances data discovery functionality, provides high-quality data curation, mediates data access via an auditable process adaptable for compliance with relevant governance requirements, provides secure data transfer, reduces the need for continued data transfer between research groups, and provides virtual data analysis workspaces. Currently, DPAU is onboarding the 44 COSMIC consortia cohort-studies [72] from 33 countries, and hopes to expand to include more studies, such as those in STROKOG [38] and other ageing studies. DPAU applies a standard data ontology to DPAU studies, with the aim to enable platform interoperability with other data initiatives including DPUK and the ADDI. DPAU aims to expand to include imaging and genetics data in addition to current data.

Table 2. Data and analysis status, and processes for data access by external researchers. Current data collection includes direct from participants, increasing availability of online data, or onboarding more member studies, as appropriate.

**Table 2:** Collaborations with clinical data.

**Initiative**	**Current Data** **Collection**	**Current Data** **Analysis**	**Data Available** **for Access**	**If available, procedure for data access**
MarkVCID	Yes	Yes	Yes	Access via: https://markvcid.partners.org/search_data_form
DiverseVCID	Yes	Yes	Yes	Contact research team via the online portal: https://dvcid-data.ucdavis.edu/portal/
Discovery	Yes	Yes	Unclear	
COMPASS-ND	Yes	Yes	Yes	Data will be made available to qualified researchers worldwide, according to data access and publication policies: https://ccna-ccnv.ca/publication-policy/
Heart Brain Connection	No	Yes	Yes	Contact the investigators
Meta VCI Map	Yes	Yes	Yes	Contact consortium leads for instructions
STROKOG	Yes	Yes	Yes	Applicant needs to submit a project proposal. Contact consortia lead for instructions.
Dementias Platform UK	Yes	Yes	Yes	Apply using our application form, all bona fide industry and academic researchers can apply for access to cohort data: https://portal.dementiasplatform.uk/Apply
Dementias Platform Australia	Yes	Yes	Yes	Submit data access application via DPAU website, subject to individual study approval: https://portal.dementiasplatform.com.au/
CADASIL Consortium	Yes	Yes	Yes	Will be determined
CADREA	Yes	Yes	No	-
AusCADASIL	Yes	Yes	Yes	Applicant will need to make a project proposal, which will require approval from the steering committee
ENIGMA	Yes	Yes	Yes	Access via: https://enigma-brain.org/enigmavis/

### 3.5 Development of international consensus criteria and guidelines

International collaboration requires the standardisation of terminology, criteria and procedures so that exchange of ideas and materials can be facilitated. A number of consensus-building and harmonisation efforts have taken place in relation to VCID. Some examples are given below.

#### 3.5.1 STandards for ReportIng Vascular changes on nEuroimaging (STRIVE)

The STRIVE initiative [73] aimed to address the issue of variable terminology in cSVD neuroimaging. The STRIVE working group consists of experts in cSVD research, particularly but not limited to neuroimaging, from around the world. STRIVE aims to clarify definitions of cSVD features on neuroimaging and to promote consistent and unbiased use of agreed-upon consensus terminology. STRIVE also provides recommendations for image acquisition and analysis. The STRIVE initiative was extended into STRIVE-2 [74] to reflect on the original terminology and update it where necessary, focusing on new information that has emerged since STRIVE-1. Of note, STRIVE-2 added quantitative imaging of brain structure and vascular function. The current manuscript also highlights unresolved issues that require further research and provides guidance for the evaluation of emerging cSVD markers and methods.

#### 3.5.2 HARmoNizing brain imaging mEthodS for vaScular contributions to neurodegeneration (HARNESS)

The HARNESS initiative [75] was established to create a framework for developing neuroimaging biomarkers of cSVD, reviewing the status of emerging neuroimaging biomarkers of cSVD, and developing and implementing standardized acquisition protocols. The 70 members of this multidisciplinary group from 29 institutions in 12 countries have participated in 11 working groups and an in-person meeting. A framework for validation was developed, followed by technical validation, biological validation and finally qualification of real-world feasibility and cost effectiveness. The validity of existing biomarkers was reviewed, with the best current evidence for lacunes, infarcts, WMH, cerebral microbleeds, atrophy, and diffusion tensor imaging being documented [76,77]. The HARNESS website disseminates standardised MRI acquisition protocols, and downloadable software packages for analyzing cSVD lesions, case report forms, and scales. The website will be periodically updated with new lesion types, acquisition parameters, and software packages. Creating a central image repository was explored but was not considered feasible due to costs involved in obtaining institutional review board approvals, legal agreements between institutions, and hosting the database.

#### 3.5.3 Framework for Clinical Trials in Cerebral Small Vessel Disease (FINESSE)

FINESSE [78] was developed to address concerns regarding trial methodology in cSVD under the auspices of the International VASCOG Society. Experts in cSVD trials were designated a particular work package: study populations, inclusion and exclusion criteria; clinical end points; cognitive testing; imaging markers; fluid biomarkers; or novel trial designs including Mendelian randomization. These working groups reviewed, discussed, and considered the literature to produce recommendations which then met whole-group consensus via a Delphi approach. The results of FINESSE included recommendations for cSVD trial design, and perspectives regarding effectiveness of currently available cardiovascular interventions in cSVD as compared to other strokes.

#### 3.5.4 Vascular Impairment of Cognition Classification Consensus Study (VICCCS)

VICCCS-1 [79] Delphi compiled responses from international VCID researchers regarding the merits and limitations of over 10 publications that proposed VCID subtype approaches and nomenclature. A 67 % agreement threshold from 98 to 153 respondents (from 27 countries) over the survey rounds resulted in redefining classification of mild and major VCID and subtypes, and identified priorities for future research. VICCCS-2 [80] Delphi evaluated VCID diagnostic assessment utility for use in clinical settings. VICCCS-2 compiled responses from 65 to 79 respondents over 6 successive survey rounds, culminating in endorsement for the standardized research use of the National Institute of Neurological Disorders-Canadian Stroke Network (NINDS-CSN) recommendations for neuropsychological and imaging assessments for VCID diagnosis. VICCCS-2 also revised diagnoses of mild and major forms of VCID based on research advances and DSM-V updated guidelines.

#### 3.5.5 Vascular Contributions to Dementia- Centre of Research Excellence (VCD-CRE) Delphi

The VCD-CRE Delphi study aims to update the earlier criteria for the diagnosis of vascular cognitive disorders (i.e., VASCOG; [81]), which have been well-validated against other diagnostic criteria [82] and have served as a standard to determine the prevalence of PSCI [83]. This update (VASCOG-2) will improve criteria usability, harmonisation, and VCID diagnostic sensitivity in accordance with research advancements. A parallel Delphi aims to develop a harmonised neuropsychological test battery for cognitive changes associated with vascular cognitive disorders by consolidating the NINDS-CSN Vascular Cognitive Impairment Harmonization Standards [84], with other harmonisation efforts [85] and introducing flexible assessment modes. The Delphi surveys will be completed by clinicians, researchers, or clinician-researchers with experience in the assessment of cognitive decline, specifically vascular cognitive disorders. Each Delphi involves three rounds of online surveys and expert meetings. Data collection is expected to be completed by December 2023. The goal is that VASCOG-2 and the harmonised neuropsychological assessment battery become the standards for future VCID diagnosis and assessment. This initiative is under the aegis of the International VASCOG Society.

## 4 Future recommendations and conclusion

This paper presents an overview of recent significant collaborative initiatives in VCID. Although our understanding and appreciation of VCID has grown immensely from these and other efforts, there is clearly a need for increased research effort in this field. One area we have identified for improvement is consistency amongst terminology and protocols in this field. Although there have been harmonisation attempts such as STRIVE, FINESSE, HARNESS, VICCCS, and the ongoing VCD-CRE Delphi there are still preferences of terminology and protocols which complicate the interpretation and comparison of data across studies.

This paper identified numerous studies which are focusing on biomarkers and pathology of cSVD. This may help to uncover the relationship between physiology and phenotype for both clinical manifestations of cSVD and cognitive decline. Future research should build on these findings to explore new diagnostic and therapeutic options.

The inclusion of risk factors in several studies in this paper has helped to understand the contributions to cSVD development and progression, and to identify individuals at risk of developing VCID. An expansion of this would be the investigation of the impact of exposure to risk factors during different periods of life. Investigating the influence of early-, mid- or late-life risk exposure could inform diagnosis and management of VCID.

While there are efforts to include diversity in VCID research, this paper also indicates the underrepresentation of participants and researchers from several backgrounds, particularly African and Asian countries, in these large-scale efforts.

We hope that this survey will help galvanize further national and international collaborative initiatives to better address the significant global health burden that is VCID.

## CRediT authorship contribution statement

**Danit G Saks:** Writing – review & editing, Writing – original draft, Project administration, Methodology, Formal analysis, Data curation, Conceptualization. **Eric E Smith:** Writing – review & editing, Writing – original draft, Methodology, Data curation, Conceptualization. **Perminder S Sachdev:** Writing – review & editing, Writing – original draft, Supervision, Methodology, Funding acquisition, Formal analysis, Data curation, Conceptualization.

## Declaration of Competing Interest

The authors declare that they have no known competing financial interests or personal relationships that could have appeared to influence the work reported in this paper.

## Appendix Supplementary materials

Image, application 1
