# Supplementary material for: National and international collaborations to advance research into vascular contributions to cognitive decline
Source: Cereb Circ Cogn Behav. 2023 Dec 14;6:100195. doi: 10.1016/j.cccb.2023.100195 (PMC10788430; doi:10.1016/j.cccb.2023.100195)
Supplement: Supplementary file 1 [file mmc1.docx]

**Supplementary**

**Acknowledgements:** The following individuals kindly provided information on the initiatives they lead or a part of: Sarah Bauermeister,^1^ Adam Bentvelzen^2^, Geert Jan Biessels^3^, Amy Brodtmann^4,5^, Rory Chen^2^, Mat J Daemen^6^, Charles DeCarli^7^, Marco Duering^8^, Israel Fernandez^9^, Myriam Fornage^10^, John Gallacher^1^, Michael D Geschwind^11^, Steven M Greenberg^12^, Masafumi Ihara^13^, Patrick Kehoe^14^, Sook-Lei Liew^15,16^, Jessica W Lo^2^, Axel Montagne^17, 18^, Jane S Paulsen^19^, Sarmi Sri^20^, Herpreet Singh^12^, Paul Thompson^15^, Joanna M Wardlaw^17, 18^, Bradford Worrall^21^.

^1^ Department of Psychiatry, University of Oxford, Oxford, United Kingdom

^2^ Centre for Healthy Brain Ageing, School of Clinical Medicine, University of New South Wales, Sydney, New South Wales, Australia

^3^ Department of Neurology, University Medical Center Utrecht, Utrecht, The Netherlands

^4^ Cognitive Health Initiative, Central Clinical School, Monash University, Melbourne, Victoria, Australia

^5^ Florey Institute of Neuroscience and Mental Health, University of Melbourne, Melbourne, Victoria, Australia
^6^ Amsterdam University Medical Center, University of Amsterdam, Pathology, Amsterdam, The Netherlands

^7^ Department of Neurology and Center for Neuroscience, University of California, Davis, California, USA

^8^ Institute for Stroke and Dementia Research, University Hospital, LMU Munich, Germany

**^9^** Stroke Pharmacogenomics and Genetics, Biomedical Research Institute Sant Pau, Sant Pau Hospital, Barcelona, Spain

^10^ Medical School and Human Genetics Center, School of Public Health, University of Texas Health Science Center at Houston, Texas, USA.

^11^ Department of Neurology, University of California San Francisco, San Francisco, California, USA

^12^ Department of Neurology, Massachusetts General Hospital, Boston, Massachusetts, USA

^13^ Department of Neurology, National Cerebral and Cardiovascular Center, Osaka, Japan

^14^ Dementia Research Group, Clinical Neurosciences, Bristol Medical School, University of Bristol, Southmead Hospital, Bristol, United Kingdom

^15^ Mark and Mary Stevens Neuroimaging and Informatics Institute, Keck School of Medicine, University of Southern California, Los Angeles, California, USA

^16^ Chan Division of Occupational Science and Occupational Therapy, Los Angeles, California, USA

^17^ UK Dementia Research Institute, Edinburgh Medical School, University of Edinburgh, Edinburgh, United Kingdom.

^18^ Centre for Clinical Brain Sciences, University of Edinburgh, Edinburgh, United Kingdom

^19^ Department of Neurology, University of Wisconsin-Madison, Madison, Wisconsin, USA

^20^ UK Dementia Research Institute HQ, London, United Kingdom

^21^ Department of Neurology, University of Virginia, Charlottesville, Virginia, USA

**Supplementary Table 1. National and International Collaboration Details**

| **Initiative** | **Principal Investigator/s** | **Key contact/s** | **Year established** | **Countries involved** | **Funding sources** | **Current/anticipated sample size** | **Website** |
| --- | --- | --- | --- | --- | --- | --- | --- |
| MarkVCID | Steven M. Greenberg, Hanzhang Lu, Gary A. Rosenberg, Danny JJ Wang, Gregory Jicha, Ronald Petersen, Joel Kramer, Jin-Moo Lee, Claudia Satizabal, Konstantinos Arfanakis | Steven M. Greenberg, Herpreet Singh | 2017 | USA (coordinating centre and 9 award sites, comprised of 16 individual medical centres) | NIH and NINDS/NIA | 2453 (MarkVCID1: 653; MarkVCID2: 1800) | https://markvcid.partners.org/ |
| DiverseVCID | Charles DeCarli, Myriam Fornage | Charles DeCarli, Myriam Fornage | 2020 | USA (12 sites/10 states) | NINDS | Current 666, anticipated 2250 | https://diversevcid.sf.ucdavis.edu/ |
| Discovery | Natalia S. Rost, and Steven M. Greenberg, | Kristin Schwab, James Meschia Rebecca Gottesman, Lisa Wruck, Karl Helmer, Alex Sherman, Hong Yu, Robert Rissman | 2019 | USA (30 sites) | NINDS, NIA | Enrolling 8000 | https://discoverystudy.org/ |
| COMPASS-ND | Eric Smith,  Simon Duchesne,  Fuqiang Gao,  Feryal Saad,  Victor Whitehead,  Cheryl R. McCreary,  Richard Frayne,  Serge Gauthier,  Richard Camicioli,  Michael Borrie, Sandra E. Black | Eric Smith | 2018 | Canada | Canadian Institutes of Health Research, via the Canadian Consortium on Neurodegeneration in Aging | 1,772 | https://ccna-ccnv.ca/compass-nd-study/ |
| Heart Brain Connection | Geert Jan Biessels, Mat J Daemen | Geert Jan Biessels, Mat J Daemen | 2013 | The Netherlands | Dutch Heart Foundation | Several clinical studies are included with a sample size varying between 20 and 500 patients | http://www.hart-brein.nl/ |
| Meta VCI Map | Geert Jan Biessels | Geert Jan Biessels, Matthijs Biesbroek, Floor de Kort | 2017 | **Europe:** The Netherlands, UK, Germany, France, Austria, **Australasia:** Korea, Hong Kong, Taiwan, Israel, Australia, Singapore  **North America:** Canada, USA | Dutch Research Council | 25000 | https://metavcimap.org/ |
| UK DRI Vascular Theme | Joanna M. Wardlaw, Axel Montagne | Sarmi Sri, Paresh Malhotra | 2019 | UK (7 centres) | Medical Research Council, Alzheimer’s Society and Alzheimer’s Research UK |  | www.ukdri.ac.uk |
| STROKOG | Perminder Sachdev | Jessica Lo | 2016 | **Europe:** UK, France, The Netherlands, Germany, Finland, Poland, Sweden, Bulgaria  **Australasia:** Republic of Korea, Australia, Singapore, Hong Kong, China  **North America:** USA.  **Africa:** Nigeria, South Africa | Vincent Fairfax Family Foundation, NHMRC project grant, NHMRC CRE | 38 member studies with over 20 000 participants | https://cheba.unsw.edu.au/consortia/strokog |
| Dementias Platform UK | John Gallacher | Sarah Bauermeister | 2014 | **Europe:**  UK, Spain, Sweden, Norway, Greece, Germany, France, Finland, Belgium, Czech Republic Ireland, Italy, Netherlands, Switzerland,  **Australasia:**  Taiwan, Hong Kong, Japan, China, Australia, Republic of Korea,  **Americas:**  USA, Canada, Brazil,  **Africa:**  South Africa | Medical Research | 3.6M | https://www.dementiasplatform.uk/ |
| Dementias Platform Australia | Perminder Sachdev | Rory Chen; Vibeke Catts | 2021 | **Europe:**  Germany, Greece, Italy,  **Australasia:**  Australia, Singapore, Taiwan, Malaysia, Philippines, China, India, Indonesia,  **Americas:**  Brazil, Puerto Rico, USA  **Africa:**  Central African Republic, Congo,  Nigeria | NIH | Nil | https://www.dementiasplatform.com.au/ |
| CADASIL Consortium | Jane S. Paulsen and Michael D. Geschwind | Kerry Ludke and Angela Gifford | 2022 | USA (12 sites) | NIA, NIH | 500 | https://cadasil-consortium.org/ |
| CADREA | Masafumi Ihara, Jay Chol Choi, Yi-Chung Lee, Sung-Chun Tang | Masafumi Ihara | 2023 | **East Asia:** Japan, Republic of Korea, Taiwan | To be explored | 1000 cases (target sample size) |  |
| AusCADASIL | Perminder Sachdev, Christopher Levi, Michael O’Sullivan, Amy Brodtmann, Beata Bajorek | Danit Saks | 2023 | Australia (5 sites) | NHMRC- (CRE) | Anticipated 150 NOTCH3 Positive and 150 controls | https://cheba.unsw.edu.au/research-projects/vascular-contributions-dementia-centre-research-excellence/auscadasil |
| STRIVE | Joanna M. Wardlaw, Martin Dichgans, Eric E. Smith, Marco Duering | Joanna M. Wardlaw, Marco Duering | 2012 | **Europe:**  Germany, The Netherlands, France, Austria, UK, Finland, Italy, Switzerland, Spain  **Australasia:**  Australia, Republic of Korea, China, Singapore  **Americas:** Canada, USA, Ecuador  **Africa:** Nigeria | Network of Centres of Excellence in Neurodegeneration (COEN) | N/a |  |
| FINESSE | Hugh S. Markus |  | 2020 | **Europe:** The  Netherlands, UK, France, Germany, Austria, Portugal, Switzerland **Australasia:**  Australia, Singapore, Republic of Korea, China,  **North America:** Canada, USA | LMU-University of Cambridge joint funding initiative | N/a |  |
| HARNESS | Eric E. Smith, Joanna M. Wardlaw | Eric E. Smith | 2018 | **Europe:** UK, The Netherlands, France, Germany, Austria, Italy, Denmark  **Australasia:** Singapore, China, Australia, **North America:** USA, Canada, | European Joint Program on Neurodegenerative Diseases |  | www.harness-neuroimaging.org |
| International Stroke Genetics Consortium | Bradford Worrall, Israel Fernandez, Stephanie Debette, Natalia Rost, Stephen Cole, Steve Kittmer | Brad Worrall, Amy Brodtmann, Matthew Pase | 2007 | **Europe:**  Switzerland, UK, Sweden, Spain, Italy, Germany, Estonia, Finland, Norway, The Netherlands, Denmark, Iceland, Ireland, Belgium, Austria, Poland  **Australasia:**  Australia, Hong Kong, China, Japan, Taiwan, India, Singapore Republic of Korea, Israel  **Americas:**  USA, Costa Rica, Chile, Argentina, Brazil, Canada,  **Africa:**  Nigeria, Zambia, Senegal, Ghana | NIH | 40000+ | www.strokegenetics.org |
| ENIGMA Stroke Recovery | Sook-Lei Liew, Paul M. Thompson |  | 2009 | **Europe:** Spain, Germany, France, Norway, Switzerland Cyprus, UK, Italy, **Australasia:**  Australia, China, New Zealand, **Americas:** Canada, USA, Brazil | American Heart Association | 2100 stroke patients from 39 research studies | https://enigma.ini.usc.edu/ongoing/enigma-stroke-recovery/ |
| VICCCS | Patrick Kehoe | Patrick Kehoe. Olivia Skrobot | 2013 | **Europe:** Finland, UK, Italy, Poland, France, Sweden, Ireland, Portugal, Russia, The Netherlands, Spain, Germany, Greece, Austria, Belgium  **Australasia:**  China, India, Israel, Japan, Australia, Singapore,  **Americas:**  Canada, USA, Trinidad,  Argentina, Brazil  **Africa:** South Africa, Nigeria | Alzheimer's Society (UK) | Approximately 300 researchers |  |
| VCD-CRE Delphi | Perminder Sachdev | Adam Bentvelzen | 2022 | Australia | NHMRC (CRE) | Delphi 1 - 70. Delphi 2 - 40. | https://cheba.unsw.edu.au/research-projects/vascular-contributions-dementia-centre-research-excellence/delphi |
| SVDs@Target | Martin Dichgans, Mark T. Nelson, Peter Rothwell, Maiken Nedergaard, Joanna M. Wardlaw, Anne Joutel, Geert Jan Biessels, Lydia Sorokin, Robert J. Oostenbrugge, Silvia Egert, Gary Randall | Karin Waegemann, Anna Kopzcak, Masayo Koide, Nicholas Klug, Shane Lyons, Alastair Webb, Iben Lundgaard, Pia Christensen, Gordon Blair, Fergus Doubal, Michael Thrippleton, Monara Angelim, Julien Ratelade, Hussein Kalakech, Valeria Domenga, Jeroen Hendrikse, Jaco Zwanenburg, Jeroen Siero, Tine Arts, Laurien Onkenhout, Hilde van den Brink, Tushar Deshpande, Jula Huppert, Erik Biessen, Walter Backes, Julie Staals, Sebastien Foulquier, Danielle Kerkhofs, Elisabeth Andre, Alfred Zollner, Claudia Pfander, Amrita Choudhary, Gabriele Wagner | 2016 | **Europe:** Germany, UK, Denmark, France, The Netherlands  **Americas:** USA | European Union’s Horizon 2020 research and innovation programme | 1650 | https://www.svds-at-target.eu/index.html |
| BRIDGET | Stephanie Debette, Helena Schmidt, Tomi Pastinen, Mark Lathrop, Hans Jörgen Grabe, M Arfan Ikram, Ian Deary, Gunter Schuman |  | 2016 | Partners: **Europe:** France, Austria, Germany, The Netherlands, The UK. **Americas:** Canada.  External collaborators: **Europe:** France  **Australasia:** Australia, Japan  **Americas:** USA, Canada | European Union Joint Programme – Neurodegenerative Disease Research | 36700 | https://bridget.u-bordeaux.fr/ |
| CHARGE | Bruce M. Psaty, Christopher J. O’Donnell, Vilmundur Gudnason, Kathryn L. Lunetta, Aaron R. Folsom, Jerome I. Rotter, André G. Uitterlinden, Tamara B. Harris, Jacqueline C.M. Witteman, and Eric Boerwinkle | Sudha Seshadri Claudia Satizabal, Jan Bressler | 2009 | **Europe:** Iceland, The Netherlands  **Americas:** USA | National Heart, Lung, and Blood Institute | Over 50 000 | https://web.chargeconsortium.com/ |
| RHU-SHIVA | Stephanie Debette and Thierry Couffinhal | Judith Thomas Crusells, Morgane Lachaize | 2020 | France (6 sites) | French National Research Agency (ANR) as part of the “Investments d’Avenir” Programme | 30 000 | https://rhu-shiva.com/en/ |

Supplementary Table 1 indicates relevant details of each initiative including investigators, year of establishment, which countries are involved, funding sources, current and/or anticipated sample size and website, if applicable. The following abbreviations were used: National Institute of Neurological Disorders and Stroke (NINDS), National Institute on Aging (NIA), National Institute of Health (NIH), Centres of Research Excellence (CRE), National Health and Medical Research Council (NHMRC).
